# Mental Imagery-Based Training to Modify Mood and Cognitive Bias in Adolescents: Effects of Valence and Perspective

**DOI:** 10.1007/s10608-016-9795-8

**Published:** 2016-08-08

**Authors:** S. Burnett Heyes, A. Pictet, H. Mitchell, S. M. Raeder, J. Y. F. Lau, E. A. Holmes, S. E. Blackwell

**Affiliations:** 1 School of Psychology, University of Birmingham, Edgbaston, Birmingham UK; 2 Department of Experimental Psychology, University of Oxford, Oxford, UK; 3 Faculty of Psychology and Educational Sciences, University of Geneva, Geneva, Switzerland; 4 Medical Research Council Cognition and Brain Sciences Unit, Cambridge, UK; 5 Oxford Centre for Human Brain Activity, University of Oxford, Oxford, UK; 6 Institute of Psychiatry, Psychology and Neuroscience, King’s College London, London, UK; 7 Department of Clinical Neuroscience, Karolinska Institutet, Solna, Sweden; 8 Mental Health Research and Treatment Center, Faculty of Psychology, Ruhr-Universität Bochum, Bochum, Germany

**Keywords:** Mental imagery, Field perspective, Observer perspective, Adolescence, Cognitive bias modification, Scrambled sentences task, Psychopathology, Cognitive training, Emotion

## Abstract

Mental imagery has a powerful impact on emotion and cognitive processing in adults, and is implicated in emotional disorders. Research suggests the perspective adopted in mental imagery modulates its emotional impact. However, little is known about the impact of mental imagery in adolescence, despite adolescence being the key time for the onset of emotional dysfunction. We administered computerised positive versus mixed valence picture-word mental imagery training to male adolescent participants (N = 60, aged 11–16 years) across separate field and observer perspective sessions. Positive mood increased more following positive than mixed imagery; pleasantness ratings of ambiguous pictures increased following positive versus mixed imagery generated from field but not observer perspective; negative interpretation bias on a novel scrambled sentences task was smaller following positive than mixed imagery particularly when imagery was generated from field perspective. These findings suggest positive mental imagery generation alters mood and cognition in male adolescents, with the latter moderated by imagery perspective. Identifying key components of such training, such as imagery perspective, extends understanding of the relationship between mental imagery, mood, and cognition in adolescence.

**Electronic supplementary material:**

The online version of this article (doi:10.1007/s10608-016-9795-8) contains supplementary material, which is available to authorized users.

## Introduction

Adolescence is a major period of life for the onset of mental health problems (Kessler et al. 1994, 2012a, b; Ormel et al. 2014; Paus et al. 2008). Understanding the mechanisms underlying emotional dysfunction and developing appropriate treatments is a priority. In adults, research has highlighted mental imagery as a key feature and contributor to emotional dysfunction across a range of psychological disorders (Holmes and Mathews 2010). Little is known about this relationship in adolescents. In adolescence, continuing maturation of basic cognitive processes (Luna et al. 2004; Dumontheil et al. 2010; Weil et al. 2013) and emotion regulation capabilities (Silvers et al. 2012), in combination with changes in motivational and social concerns (Crone and Dahl 2012; Geier and Luna 2009; Steinberg 2008), may render individuals more vulnerable to emotional (including imagery) symptoms. Conversely, these same developmental features suggest adolescents may benefit from appropriately targeted interventions (Burnett Heyes et al. 2013). In the current study, we investigated the impact of generating emotional mental imagery on mood and cognition in adolescents.

Mental imagery, defined as the experience of perception in the absence of matching sensory input (Kosslyn et al. 2006), is hypothesised to contribute to emotional dysfunction across a range of psychological disorders (Holmes and Mathews 2010). Correlational evidence shows individuals with depression experience an excess of intrusive negative imagery, and difficulties in generating positive mental imagery (Weßlau and Steil 2014; Holmes et al. 2016a, b). In social phobia, intrusive, negative third-person perspective mental imagery during social interactions (e.g. of self blushing bright red) causes distress and negatively impacts social performance (Hirsch et al. 2003). These observations suggest an association between mental imagery and emotional and cognitive processes in emotional disorders.

Experimental evidence lends weight to the notion that mental imagery has a causal, not just correlational, relationship with emotion. Studies with non-clinical, dysphoric, depressed and socially-anxious adult samples show that deliberately generating mental imagery in response to emotionally valenced cues (e.g. verbal stories or picture-word pairs) gives rise to congruent changes in positive, depressed or anxious mood (Holmes et al. 2006, 2008b; Pictet et al. 2011; Stopa et al. 2012). In contrast, verbal processing of the same training stimuli does not give rise to comparable changes in mood (Holmes and Mathews 2005; Holmes et al. 2008b), and may even lead to mood worsening (Holmes et al. 2006, 2008a, 2009).

Generating mental imagery can also induce changes in cognitive processing, for example, in the interpretation of ambiguous information. Information processing accounts of emotional disorders highlight cognitive factors including interpretation bias that cause or maintain symptoms (Mathews and MacLeod 2002). Anxious and depressed participants tend to interpret emotionally ambiguous information in a negative manner (Mathews and MacLeod 2005; Muris and Field 2008; Wisco and Nolen-Hoeksema 2010; Orchard et al. 2015), with negative interpretation bias predicting associated symptomatology in adults (Kleim et al. 2014; Woud et al. 2014). Generating mental imagery of a particular emotional valence causes congruent changes in interpretation bias in non-clinical and dysphoric adults (Holmes et al. 2009; Pictet et al. 2011). Bias change can take place following a single session of mental imagery (Holmes et al. 2009) and persist 24 h later (Pictet et al. 2011).

In adolescent samples, changes in interpretation bias have been induced by cognitive training procedures (often termed Cognitive Bias Modification for interpretation, or CBM-I) incorporating a mental imagery element (Lau et al. 2011, 2012, Lothmann et al. 2011). Adolescent participants trained to interpret verbal material in a positively-valenced manner selected more positive interpretations than negative interpretations, whereas those trained to interpret material in a negatively-valenced manner showed effects in the opposite direction. In a subset of the above studies, participants in the positive CBM-I condition additionally showed a decrease in self-reported impact of recent stressful life events and responses to a laboratory stressor. Extending CBM-I with imagery to a sample of anxious adolescent patients, similar changes in interpretational style were found (Fu et al. 2013). Thus, these training procedures developed in adults have shown some efficacy in boosting positive interpretations in child and adolescent samples. However, the underlying mechanisms are not fully understood.

What are the components of mental imagery that give rise to its impact on mood and cognition? One candidate is mental imagery perspective. Mental imagery can be generated from a field (first person) perspective or from an observer (third person) perspective. There is evidence for individual differences in preferred imagery perspective (Robinson and Swanson 1993; Vella and Moulds 2014). Adults and adolescents with depression show greater use of the observer perspective, particularly for positive memories and events, and adults with social anxiety report intrusive negative observer perspective imagery of social interactions (Bergouignan et al. 2008; Kuyken and Howell 2006; Lemogne et al. 2006; Williams and Moulds 2007; see also Nelis et al. 2013). Observer perspective imagery is associated with reduced sensory and emotional reliving and reduced self-relevance (Berntsen and Rubin 2006; Nigro and Neisser 1983). Non-clinical adult participants instructed to generate field perspective imagery in response to positive cues showed greater changes in mood than did participants in an observer perspective condition (Holmes et al. 2008a), although Nelis et al. (2012) did not replicate this effect, suggesting the need for further investigation. Taking an observer perspective is hypothesised to dampen mood, whereas taking a field perspective is hypothesised to facilitate emotional processing (Berntsen and Rubin 2006; Holmes et al. 2008a; McIsaac and Eich 2002; Nigro and Neisser 1983). As such, explicit instruction to adopt a field perspective is hypothesised to enhance emotional impact in adults and adolescents alike. Furthermore, evidence from the cognitive developmental literature indicates that the ability to deliberately take other perspectives (cognitive perspective-taking ability) is still developing during adolescence (Dumontheil et al. 2010). Also showing extended development during adolescence are other cognitive abilities that would support deliberate, sustained adoption of a particular perspective (i.e. executive functions such as working memory; Luna et al. 2004), and awareness of the extent to which a particular perspective was being adhered to (i.e. metacognitive accuracy; Weil et al. 2013). Together, these lines of converging evidence suggest adolescents will need more guidance than adults to achieve the same level of success in adopting a particular perspective. That is, for adolescent participants, explicit guidance on how to adopt a field versus observer perspective may enhance the impact of field perspective imagery training on mood and cognition. This was evaluated in the current study.

This study used a mental imagery generation procedure in which participants were instructed to repeatedly generate positive or mixed valence field or observer perspective mental imagery in response to a set of training stimuli (Blackwell et al. 2015; Holmes et al. 2008b; Pictet et al. 2011). Stimuli were ambiguous pictures paired with disambiguating verbal captions (Pictet et al. 2011; Holmes et al. 2008a, b). For example, a participant presented with a picture of a rain cloud in combination with the word “refreshing” may imagine the pleasant feeling of cool rain on a hot day. The same picture paired with the phrase “cold and wet” may lead to the participant to generate more negatively valenced imagery. Participants viewed picture-word pairs suggesting either consistently positive resolutions (positive imagery group), or a mixture of positive and negative resolutions (mixed imagery group). Within each valence group, each participant underwent field and observer perspective imagery training on separate days (field and observer perspective conditions).

The picture-word mental imagery generation procedure, adapted from previous studies (Pictet et al. 2011; Holmes et al. 2008a, b), was optimised for adolescents. In adolescence, peer relationships assume heightened importance for social and personal development (Bagwell and Schmidt 2013; Lorme et al. 2013; Nelson et al. 2005), and social goals such as reputation and friendship can be an important motivating factor, as well as a source of distress (Davey et al. 2008; Gullone and King 1993). Therefore, in order to target key sources of concern in adolescent participants, we increased the proportion of picture-word stimuli suggesting social situations involving peers (see Table 1). For example, a participant presented with a picture of a smartphone in combination with the phrase “funny text” [i.e. SMS] may imagine a joke shared with a friend, whereas the same picture paired with “ignoring me” may prompt negative imagery. Second, to maximise task engagement, the number of picture-word cues was reduced in number, while on each trial, longer time was allowed to generate mental imagery—in view of evidence showing development during adolescence in imagery generation speed (Kosslyn et al. 1990). In view of the experimental nature of the adolescent imagery training manipulation, we recruited non-clinical participants. Given the novelty of this topic, we restricted our sample to a single gender to reduce within-sample variance and enable optimisation of imagery training stimuli content. Participants were all male due to an opportunity sample from a boys’ school. Participants rated positive and negative mood before and after imagery generation and completed three measures of cognitive (interpretation) bias.

**Table 1:** Examples of picture-word stimuli used in the imagery training task

Picture	Accompanying caption in positive valence imagery condition	Accompanying negative caption in mixed valence imagery condition
Smartphone	“Funny text” [i.e. SMS]	“Ignoring me”
Facebook screenshot	“Friend request”	“Unfriended”
Football game	“Great shot”	“Own goal”
Classroom	“Good test result”	“Exam stress”
Alarm clock	“Lie in”	“Late again”
School bag	“Found it”	“Can’t find it”
School play	“Great performance”	“Forgot my lines”
Board game	“Winner”	“Cheat”
Girls laughing	“Friends”	“Laugh at me”
Snowball fight	“Lively game”	“Not fair”

Measures of interpretation bias were as follows. First, participants rated pleasantness of a set of ambiguous photograph stimuli before and after imagery generation. This measure has shown training effects in previous imagery generation studies in adults (Holmes et al. 2008, b; Pictet et al. 2011). Second, participants completed a novel scrambled sentences task (SST) adapted from previous versions (Standage et al. 2010; Watson and Friend 1969; Wenzlaff and Bates 1998), after imagery generation. An SST has been used as an outcome measure of interpretive bias in both experimental (Holmes et al. 2009) and clinical (e.g. Blackwell et al. 2015) studies with adult participants. We constructed an SST version that was suitable for adolescent participants and that can be administered twice, following separate field and observer perspective imagery sessions. Finally, participants completed a computerised recognition task (CRT), which has been widely used in adolescent CBM-I studies (e.g. Lothmann et al. 2011; Lau et al. 2012; Fu et al. 2013), after imagery generation. The CRT was adapted to create two separate versions, for administration following separate field and observer perspective sessions.

We predicted greater increases in positive mood and fewer negative interpretations following positive than mixed imagery training. Negative interpretations were operationalized as relatively low pleasantness ratings of ambiguous photographs, negative bias score on the SST, and endorsing negative interpretations on the CRT. Further, we predicted that the impact of positive imagery generation would be greater following field than observer perspective imagery.

## Method

### Participants

60 individuals (male; mean age = 13.8, SD = 1.0; age range 11.9–16.3 years) were recruited via letters sent home from school (N = 45; of which N = 40 private school and N = 5 state school) or advertisements in the local community (N = 15). An information sheet given to parents specified that individuals with “a history of mood or anxiety problems” were not eligible to take part. The study was approved by the University of Oxford Central University Research Ethics Committee and was carried out in accordance with the provisions of the World Medical Association Declaration of Helsinki. The current sample size of 60 was determined based on Pictet et al. (2011) which used a picture-word imagery generation task with approximately N = 30 per valence group.

### Design

Participants were randomly assigned to receive two sessions of either positive or mixed imagery training. Each participant completed both field and observer perspective imagery sessions on separate days, with perspective order counterbalanced within valence groups.

### Overview of the Procedure

In each session, participants first completed baseline measures of mood and one measure of interpretation bias, followed by mental imagery instructions and the picture-word imagery generation task. After training, participants repeated mood and interpretation bias measures and completed two further measures of interpretation bias. See Figure S1 (Supplementary Material) for a timeline of the experimental sessions.

### Materials

#### Positive and Negative Affective State

Positive and negative affective state was assessed using emotion words from the Positive and Negative Affect Schedule (PANAS-X; Watson and Clark 1994). Each emotion word was rated on a 5-point scale (“not at all” to “extremely”), using the state instructions (“Indicate to what extent you feel this way right now”). To create a brief measure that was engaging for participants and could be administered repeatedly on a laptop, 21 emotion words were selected, in total fourteen negative (seven fear, seven sad) and nine positive items. An E-prime programme randomly selected six negative (three sad, three fear) and four positive words (i.e. ten words in total). Ten words per PANAS administration was selected as a satisfactory compromise between brevity (maximising task engagement) and variance (maximising operating range/power). An equal number of sad and fear words was selected since positive imagery training effects have been reported on both anxious mood (Holmes et al. 2008a, b) and depressive symptoms (Lang et al. 2012) in adults. For analyses of PANAS data, dependent variables were t1–t2 standardised residuals representing change in mean positive and mean negative mood ratings from t1 to t2 correcting for any differences at t1 (Jacob et al. 2013).

#### Pleasantness Ratings of Picture Stimuli

Pleasantness ratings were given in response to 40 picture stimuli as a measure of interpretation bias. The set consisted of 20 “novel” and 20 “familiar” pictures. “Novel” stimuli were selected at random each time the task was administered from a set of 40 pictures selected from previous studies that did not appear in the imagery generation task. “Familiar” stimuli were chosen at random each time the task was administered from the set of 75 pictures used in the picture-word imagery generation task. This factor was included to investigate generalisation of training effects. An E-prime task programme selected the pictures and presented them in a random order without words against a black background for 1500 ms. Participants rated the subjective emotional valence of each picture from 1(“extremely unpleasant”) to 9 (“extremely pleasant”). For analyses of pleasantness data, the dependent variable was t1–t2 standardised residuals representing change in mean ratings from t1 to t2 correcting for any differences at t1 (Jacob et al. 2013). In addition, to investigate generalisation of training effects, a stimulus Novelty factor (two levels: Familiar, Novel) was included.

#### Mental Imagery Instructions

A standardised mental imagery instruction script was adapted from Pictet et al. (2011). First, mental imagery was defined as the experience of “seeing with the mind’s eye, hearing with the mind’s ear, and so on”, using Kosslyn et al. (2006) definition, i.e., in any sensory modality. Participants were prompted to give their own definition of mental imagery, to check comprehension. The experimenter next gave either field or observer perspective instructions according to condition assignment (field: “Right now, we’re interested in mental imagery that you imagine seeing through your own eyes, happening to you, as if you are actively involved”; observer: “Right now, we’re interested in mental imagery that you imagine from a detached point of view, as if you’re observing yourself in a situation—seeing yourself from above, or from the outside, from behind or in front, or as if you’re watching yourself on TV”), which participants were prompted to briefly practice by imagining they were in their bedroom (field), or imagining they could see themselves in the testing room (observer). All participants were then given a standardised imagery generation practice task of imagining seeing, holding and cutting a lemon (field), or imagining they could see themselves doing so (observer). Within this practice task, participants were familiarised with paper versions of vividness and perspective rating scales (for further details, see next section). Finally, participants were given practice in generating field or observer perspective mental imagery in response to two (both positive, or one positive and one negative) picture-word cues presented on paper.

#### Picture-Word Cues to Generate Mental Images

Participants generated field or observer perspective mental imagery in response to 79 picture-word cues on a laptop (4 practice and 75 training). The stimuli comprised pictures selected from previous studies (Pictet et al. 2011), created for the study by the authors, or downloaded from the internet.

Each picture was paired with a word or short phrase intended, when paired with the relevant picture, to create a positive emotional resolution (e.g. a picture of a hand holding a mobile phone and the phrase “funny text” [i.e. SMS]) or a negative emotional resolution (e.g. the same picture paired with the phrase “ignoring me”; see Table 1). In the positive condition, all pictures were paired with captions that suggested a positive resolution. In the mixed condition, the computer program randomly paired half of the pictures with captions that suggested a negative resolution and half with captions that suggested a positive resolution. All participants saw the same pictures, but the proportion with positive/negative valence differed according to condition assignment. Approximately 50 % of the stimuli suggested a social situation.

Picture-word cue stimuli were presented using E-Prime (Schneider et al. 2002) on a laptop. Instructions on the screen for 1000 ms stated “imagine the combination of the next picture and word as if you were actively involved [field]/from a detached perspective [observer]”. Four practice and 75 training trials were presented in randomised order, the latter in blocks of 5.

For each picture-word cue participants were instructed to shut their eyes and generate a mental image that combined the picture with the word. Captions were displayed in white against a black background, centrally beneath the relevant picture for 4500 ms, followed by a 1000 ms beep. At or after the beep, participants opened their eyes and rated imagery vividness on a scale from 1 (“not at all vivid”) to 5 (“extremely vivid”). After every practice trial, and after every five training trials, participants were also prompted to rate the extent to which the perspective of their mental image corresponded to field or observer instructions, on a scale from 1 (“not at all involved/detached”) to 5 (“extremely involved/detached”), and to talk to the experimenter, to check task compliance. Here, the experimenter prompted participants to describe (if they could) an image from the most recent block, and gave positive verbal feedback for descriptions which (a) made use of both the picture and caption, (b) were consistent with field or observer perspective imagery (McIsaac and Eich 2002) and (c) described imagery content suggestive of sensory image-based (rather than language-based) processing (Holmes and Mathews 2010).

#### Filler Task to Equalize Mood

Participants listened to six 40 s classical music extracts (Holmes et al. 2008b, 2009; Pictet et al. 2011). The filler task is administered to return mood to baseline levels following imagery generation, prior to completing measures of interpretation bias. Thus, any differences in interpretation bias scores would not be attributable to affective carryover from mental imagery (i.e., mood priming). To promote task compliance, participants rated how pleasant they found each musical extract on a scale of 1 (“extremely unpleasant”) to 9 (“extremely pleasant”).

#### Scrambled Sentences Task

A novel scrambled sentences task (SST) was administered to measure interpretation bias (see Table S1, Supplementary Material). Participants unscrambled a list of 20 scrambled sentences, using five out of the six displayed words, within a time limit of 4 min. This was completed under cognitive load (holding in mind a 4-digit number). Each sentence (e.g. “will people my mistakes notice talents”) could be unscrambled to form a positive resolution (“people will notice my talents”) or a negative resolution (“people will notice my mistakes”). A negativity score was generated by calculating the proportion of sentences completed correctly with negative valence; this was used for descriptive and inferential statistics. Two versions of the SST, each containing different stimuli, were used in the current study (i.e. for field and observer perspective imagery sessions), with SST version order counterbalanced across valence and perspective conditions. The SST provides a pen-and-paper measure of interpretation bias.

##### Construction and Validation of the Novel Scrambled Sentences Task

We adapted the task from adult SST versions (Standage et al. 2010; Watson and Friend 1969; Wenzlaff and Bates 1998) resulting in an adolescent-appropriate SST. In view of the imagery training procedure’s emphasis on social situations involving peers, our novel SST comprised statements reflecting general and social anxiety-related concerns. Stimuli were constructed using statements from the Kiddie Schedule for Affective Disorders Present and Lifetime Diagnostic interview (K-SADS-PL; Kaufman et al. 1997), the Fear of Negative Evaluation Scale, and items from the adult social anxiety SST (Standage et al. 2010). All scrambled sentence stimuli were written in the first person. Forty scrambled sentence stimuli were developed (see Table S1, Supplementary Material) resulting in two versions of the SST. A random subset of 20 of these stimuli was validated in a pilot sample of N = 60 young adult controls [mean (SD) age = 20.1 (.92); 30 male]. In this pilot sample, negativity scores on the novel SST were correlated with scores on the Liebowitz Social Anxiety Scale (*r* = .350, *p* = .004; Liebowitz 1987) and with scores on the trait State-Trait Anxiety Inventory (*r* = .414, *p* ≤ .001; Spielberger 1989), suggesting sensitivity of our novel SST to general and social anxiety-related negative interpretation bias.

#### Computerised Recognition Test

A computerised recognition test (CRT) based on Mathews and Mackintosh (2000) and adapted from Lothmann et al. (2011) was administered in E-prime. The task comprises a series of 10 (from a set of 20) ambiguous social scenarios displayed in text on the screen. Participants read each description and complete a neutral word fragment. For example: “The park: You are walking through the park on your way home from having your hair cut. As you walk past some girls, you see that they are *wh*-*sp*-*ring*”. After participants had read all 10 scenarios they completed a “recognition test” to assess interpretation bias. The title of the situation (“The park”) was presented at the top of the screen along with four sequentially-presented recognition statements. Two sentences comprised “targets” depicting a positive (“The girls start to whisper because they think you look good with your new haircut”) or a negative (“The girls start to whisper because they think your new haircut looks terrible”) interpretation. Two sentences comprised “foils”, i.e., valenced statements that included information not explicitly given in the ambiguous situations. Participants were instructed to rate the similarity of each recognition statement to the material presented during the test phase, on a scale from 1 (very different in meaning) to 4 (very similar in meaning). As in previous studies, foils were included to investigate whether training effects specifically altered interpretational style (that were contained in targets) or whether training facilitated general response bias to valenced material (i.e., the tendency to give higher similarity ratings to positive or negative information congruent with the training condition). Two versions of the CRT were administered in the current study (once per session—i.e. once following field perspective imagery, and once following observer perspective imagery), with version order counterbalanced across valence and perspective conditions. Inferential statistics were conducted investigating effects of the within-subjects factors recognition statement valence (Positive, Negative) and recognition statement type (Target, Foil) on similarity ratings.

### Questionnaire Measures

#### Social Anxiety Scale for Adolescents

The social anxiety scale for adolescents (SAS-A; La Greca and Lopez 1998) is a brief self-report measure of social anxiety. Eighteen social anxiety statements are rated on a 5-point scale (true for you “not at all” to “all of the time”). The scale consists of three interrelated subscales and shows good internal consistency and test–retest reliability, with evidence for construct and concurrent validity (Inderbitzen-Nolan and Walters 2000; La Greca 1998; La Greca and Lopez 1998).

### Procedure

Written informed consent was obtained from a parent/guardian of all participants. Participants recruited via school were tested in a quiet room in school. Participants recruited via the community were tested in a quiet room at the University.

Participants were pseudo-randomly assigned to receive either positive or mixed imagery training (N = 30 per group). For each testing site, we created a randomisation scheme using the random function in Matlab such that roughly equal numbers of participants from each site were assigned to each valence group. Within groups, participants were pseudo-randomly assigned to complete field or observer perspective imagery in session 1 and the converse in session 2 such that perspective order was counterbalanced within imagery training group. This was implemented by manually constructing a counterbalancing scheme prior to testing. Sessions 1 and 2 were completed mean (SD) 4.02 (3.00) days apart (range 1–14 days).

At the start of session 1, participants completed the SAS-A (see Figure S1). Then, for each session, participants gave baseline ratings of current positive and negative mood [t1 PANAS] and Pleasantness ratings of picture stimuli [t1 Pleasantness]. Participants then completed the standardised imagery instruction and practice procedure, followed by the computerised imagery training task in which either positive or mixed field or observer perspective imagery was generated depending on group and condition order assignment. Subsequently, participants repeated the PANAS [t2 PANAS], completed the music filler task, completed a final PANAS [t3 PANAS] and Pleasantness ratings task [t2 Pleasantness], followed by two further measures of interpretation bias, the SST and CRT (see Figure S1).

### Analysis

Randomisation checks were conducted using independent samples *t* tests to identify any differences across groups in SAS-A. Subsequently, data analysis focused on establishing the effects of positive versus mixed and field versus observer imagery generation on mood (PANAS) and cognitive bias measures (Pleasantness, SST, CRT) using ANOVA. SAS-A was included as a covariate in analyses of SST and CRT data to control for variance due to pre-existing social anxiety on these two measures which were administered at one time-point (post-training) only. ANOVA was conducted on vividness ratings elicited during the imagery generation task to identify any differences between valence and perspective conditions that might contribute to interpreting observed effects. The analogous analysis could not be conducted for perspective ratings as an error in the task code meant perspective ratings were not saved for the observer condition. The following data were missing due to interruption of the testing session or equipment failure: 12 PANAS measurements, 6 Pleasantness ratings, 3 SST negativity scores, 15 CRT bias scores and 11 vividness scores (see Table 2). One data point was missing for participant exact age.

**Table 2:** Participant characteristics and effects of training

	Positive valence imagery group (N = 30)			Mixed valence imagery group (N = 30)		
	Mean	SD	N	Mean	SD	N
*Baseline data*						
Age	13.8	1.01	29	13.8	1.02	30
SAS-A	46.6	11.0	30	43.5	12.3	30
*Mood data*						
t1 PANAS positive						
Field	3.32	.884	29	3.25	.812	30
Observer	3.13	.838	30	3.15	.665	30
t2 PANAS positive						
Field	3.45	.880	29	3.18	.938	30
Observer	3.38	.754	30	3.05	.690	30
t3 PANAS positive						
Field	3.44	.758	27	3.22	.953	30
Observer	3.38	.873	30	3.19	.920	29
t1 PANAS negative						
Field	1.62	.631	29	1.60	.469	30
Observer	1.65	.616	30	1.62	.640	30
t2 PANAS negative						
Field	1.59	.637	29	1.63	.556	30
Observer	1.58	.673	30	1.60	.509	30
t3 PANAS negative						
Field	1.53	.552	27	1.61	.623	30
Observer	1.46	.638	30	1.59	.589	29
*Cognitive bias data (1)*						
*Pleasantness ratings*						
t1 Pleasantness						
Field (total)	5.57	.538	29	5.29	.459	30
Field (familiar)	5.70	.636	29	5.41	.520	30
Field (novel)	5.43	.576	29	5.17	.509	30
Observer (total)	5.69	.514	30	5.24	.660	30
Observer (familiar)	5.90	.517	30	5.33	.684	30
Observer (novel)	5.47	.621	30	5.14	.706	30
t2 Pleasantness						
Field (total)	5.80	.653	27	5.32	.582	30
Field (familiar)	6.01	.745	27	5.38	.690	30
Field (novel)	5.57	.689	27	5.26	.608	30
Observer (total)	5.61	.622	28	5.29	.523	30
Observer (familiar)	5.82	.724	28	5.33	.664	30
Observer (novel)	5.39	.613	28	5.26	.517	30
*Cognitive bias data (2)*						
SST negativity						
Field	.265	.174	28	.374	.212	29
Observer	.299	.185	30	.314	.165	30
*Cognitive bias data (3)*						
CRT similarity						
Field (Neg targets)	2.45	.460	24	2.42	.344	25
Field (Pos targets)	2.35	.430	24	2.50	.460	25
Field (Neg foils)	2.60	.351	24	2.61	.376	25
Field (Pos foils)	2.60	.346	24	2.47	.373	25
Observer (Neg targets)	2.39	.437	27	2.41	.362	29
Observer (Pos targets)	2.56	.383	27	2.49	.397	29
Observer (Neg foils)	2.61	.396	27	2.52	.356	29
Observer (Pos foils)	2.44	.407	27	2.58	.364	29
*Vividness of imagery during imagery generation task*						
Field	3.69	.451	23	3.57	.471	29
Observer	3.71	.548	28	3.48	.565	29

t1 PANAS and t1 Pleasantness data were collected prior to imagery training. t2 PANAS data were collected immediately after imagery training. t3 PANAS and t2 Pleasantness data were collected following completion of the music filler task. See “Method” section for detailed protocol

*SAS*-*A* social anxiety scale for adolescents, *PANAS* positive and negative affect schedule, *SST* scrambled sentences task, *CRT* computerised recognition test

## Results

### Randomisation Checks

Comparisons of characteristics of participants assigned to each imagery valence group revealed no significant differences at the start of session 1 in SAS-A [*t*(58) = 1.02, *p* = .312].

### Positive Affect Following Imagery Training

**Fig. 1:**
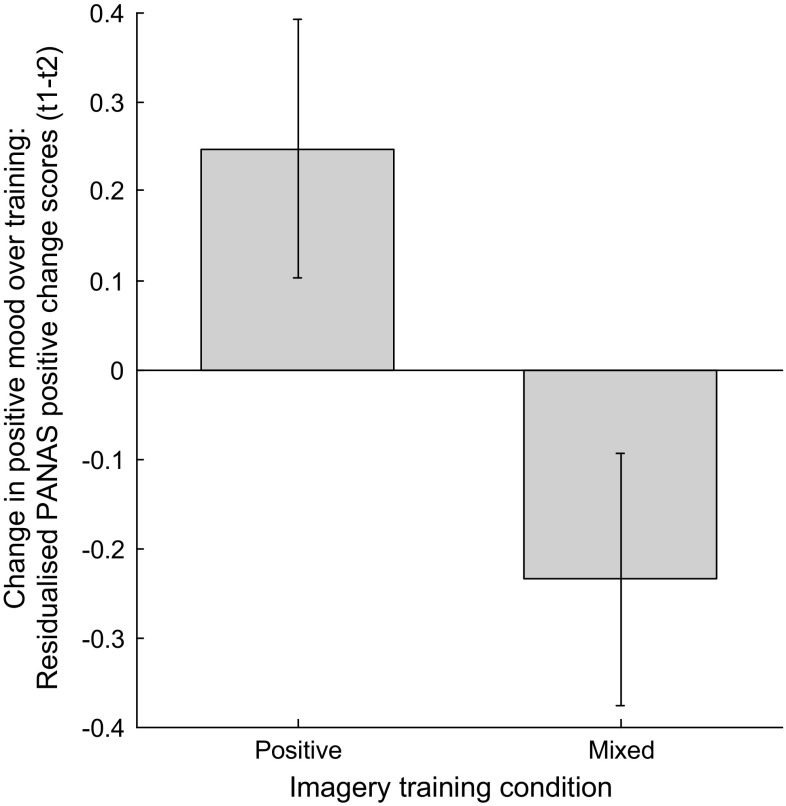
Greater increase in positive mood following positive than mixed imagery. Graph shows estimated marginal means for the main effect of imagery valence on residualised change in PANAS positive mood scores pre to post-imagery (t1 to t2). *Error bars* represent ±1 standard error of the mean. See Table 2 for raw descriptives

Residualised PANAS positive mood change scores from t1 to t2 were analysed using ANOVA with between-subjects factor Valence (Positive, Mixed) and within-subjects factor Perspective (Field, Observer). As predicted, there was a significant effect of Valence [*F*(1,57) = 5.66, *p* = .021, *ηp* ^2^ = .024; Fig. 1], indicating greater increase in positive affect in the Positive compared to the Mixed Valence condition. There was no main effect of Perspective [*F*(1,57) = .002, *p* = .963], and no interaction between Valence and Perspective [*F*(1,57) = 1.42, *p* = .239]. For PANAS positive raw mood scores at each time-point, see Table 2.

### Negative Affect Following Imagery Training

Residualised PANAS negative mood change scores were analysed in a similar manner. There was no main effect of Valence [*F*(1,57) = .620, *p* = .434] or Perspective [*F*(1,57) = .003, *p* = .957], and no interaction [*F*(1,57) = .007, *p* = .935].

### Pleasantness Ratings of Pictures Following Imagery Training

**Fig. 2:**
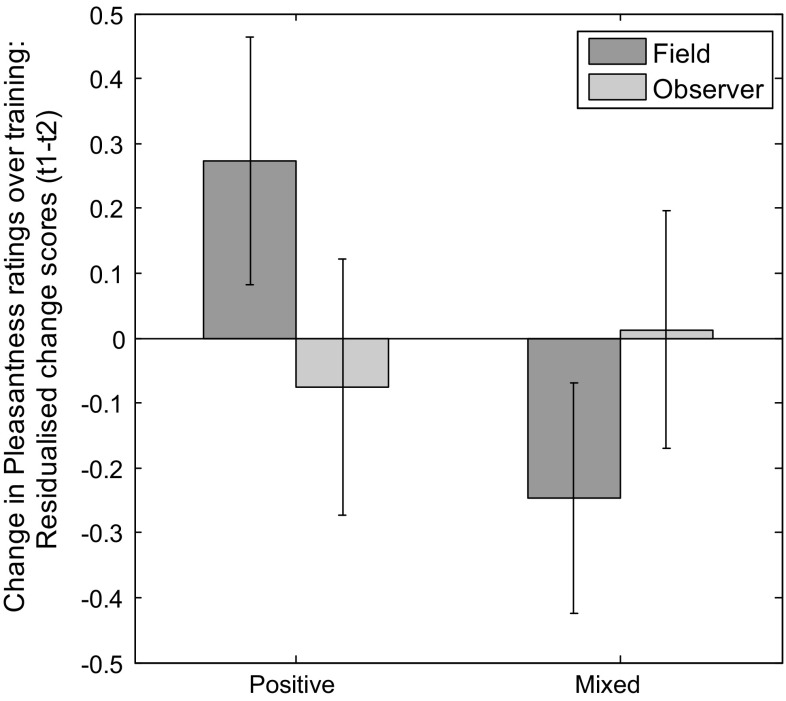
Greater increase in pleasantness ratings following positive than mixed imagery generated from a field but not observer perspective. Graph shows estimated marginal means for the interaction between imagery valence and perspective on residualised change in pleasantness ratings of picture stimuli pre to post-imagery (t1 to t2). *Error bars* represent ±1 standard error of the mean. See Table 2 for raw descriptives

Residualised pleasantness rating change scores were analysed in a similar manner. As predicted, the interaction between Valence and Perspective was significant [*F*(1,54) = 4.70, *p* = .035, *ηp* ^2^ = .080; see Fig. 2]. There was no main effect of Valence [*F*(1,54) = 1.61, *p* = .210], Perspective [*F*(1,54) = .102, *p* = .750] or Novelty [*F*(1,54) = .018, *p* = .895].

Paired samples *t* tests comparing total Pleasantness scores collapsed across Familiar and Novel stimuli confirmed a greater increase in pleasantness ratings in the Positive compared to the Mixed Valence condition for Field [*t*(26) = 2.86, *p* = .008, *d* = 1.12] but not Observer [*t*(29) = .371, *p* = .713]. Interactions between Valence and Novelty, Perspective and Novelty, and between Valence, Perspective and Novelty were non-significant [*F*(1,54) = 3.44, *p* = .069; *F*(1,54) = .003, *p* = .959; *F*(1,54) = .138, *p* = .711].

### Mood After the Filler Task

To check whether mood was comparable across valence groups and perspective conditions prior to administration of cognitive bias measures, *t*3 PANAS positive and *t*3 PANAS negative scores were analysed in two separate ANOVAs with between-subjects factor Valence and within-subjects factor Perspective. There were no significant effects in either analysis [*t*3 PANAS positive: Valence: *F*(1,54) = .446, *p* = .507; Perspective: *F*(1,54) = 1.54, *p* = .220; Valence × Perspective: *F*(1,54) = .025, *p* = .876; *t*3 PANAS negative: Valence: *F*(1,54) = .523, *p* = .472; Perspective: *F*(1,54) = 1.31, *p* = .258; Valence × Perspective: *F*(1,54) = .593, *p* = .445], indicating that Valence groups and Perspective conditions were comparable on mood prior to administration of cognitive bias measures.

### Scrambled Sentences Negativity Following Imagery Training

**Fig. 3:**
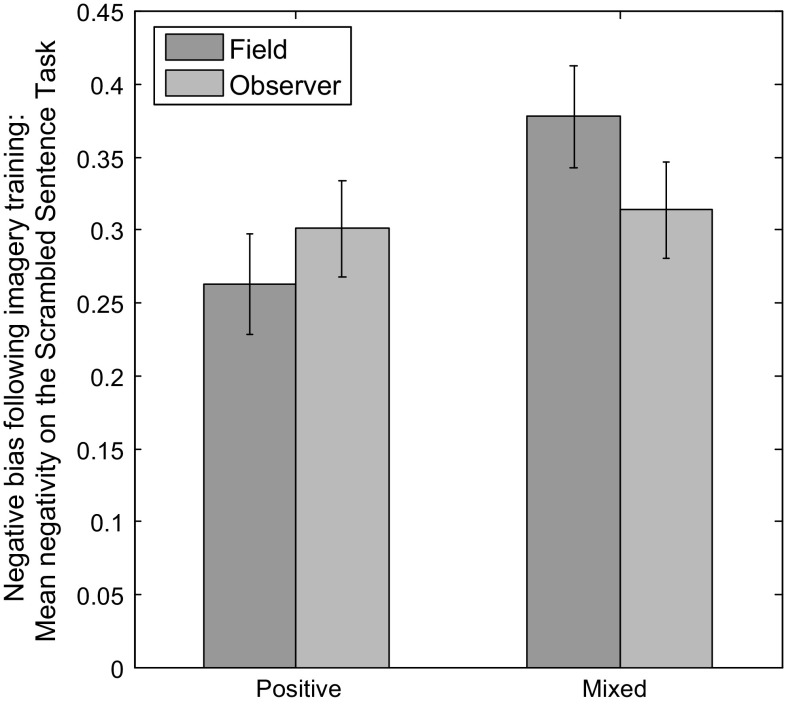
Lower scrambled sentence task negative bias following positive than mixed imagery generated from a field but not observer perspective. Graph shows estimated marginal means for the interaction between valence and perspective on scrambled sentence task negativity score after controlling for total score on the Social Anxiety Scale for Adolescents. *Error bars* represent ±1 standard error of the mean. See Table 2 for raw descriptives

SST negativity scores were analysed in an ANCOVA with between-subjects factor Valence, within-subjects factor Perspective and covariate SAS-A total score. We found the predicted main effect of Valence [*F*(1,55) = 2.83, *p*_1-tailed_ = .049, *ηp* ^2^ = .049] and predicted interaction between Valence and Perspective [*F*(1,55) = 2.95, *p*_1-tailed_ = .046, *ηp* ^2^ = .051].^1^ Post hoc *t* tests comparing SST negativity across Valence groups for each of the two Perspective conditions showed greater negativity scores in Mixed compared to Positive Valence condition for Field [*t*(56) = 2.12, *p* = .038, *d* = .558] but not Observer [*t*(58) = .326, *p* = .746] (Fig. 3). There was no main effect of Perspective [*F*(1,55) = .772, *p* = .383] or SAS-A total score [*F*(1,55) = 2.65, *p* = .110] and no interaction between Perspective and SAS-A total score [*F*(1,55) = 1.03, *p* = .314].

### Computerised Recognition Task Bias Score Following Imagery Training

Rated similarity for negative and positive targets and foils on the CRT were analysed using ANCOVA with between-subjects factor Valence, within-subjects factors Perspective, recognition statement valence and recognition statement type, and covariate SAS-A total score. There were no significant effects (all *p* > .1; see Table 2).

### Vividness of Mental Imagery Across Valence and Perspective Conditions

Vividness of mental imagery generated during the imagery generation task was analysed using ANOVA with between-subjects factor Valence and within-subjects factor Perspective. This revealed a marginal main effect of Perspective (*F*_1,48_ = 2.95, *p* = .092, ηp^2^ = .058), no effect of Valence (*F*_1,48_ = .040, *p* = .841) and no interaction between Valence and Perspective (*F*_1,48_ = 1.14, *p* = .290; see Table 2).

## Discussion

This study was conducted as a first experimental investigation of the impact on mood and interpretation bias in adolescents of generating emotionally valenced mental imagery from field and observer perspectives. Participants assigned to positive imagery training received a mood boost and gave more positive interpretations of ambiguous stimuli. Effects of positive imagery training on interpretations of ambiguous information were greater when participants generated imagery from a field than an observer perspective. Overall, results show an impact of positive mental imagery generation on mood and cognition (interpretation specifically), and highlight the moderating effects of imagery perspective.

### Increase in Positive Mood Following Positive Imagery Training

Participants in the positive imagery condition showed a greater increase in positive mood than did participants in the mixed imagery condition, in line with findings from previous studies in adults (Holmes et al. 2008b; Pictet et al. 2011). Recently, a number of studies have sought to modify mood in children and adolescents using cognitive bias modification for interpretations (CBM-I) procedures. While the format of the imagery training used in the current study is different to that of existing CBM-I protocols, conceptually they involve similar processes. Both involve resolving the ambiguity of a stimulus in a particular direction, which is then reinforced through multiple trials of repetitive learning of this ‘rule’. In Lothmann et al. (2011), male (but not female) adolescents aged 13–17 years showed a change in positive mood following a computerised word fragment completion CBM-I procedure. Another study showed a change in self-reported anxious mood in 6–11 year olds repeatedly reinforced for selecting negative or benign interpretations of ambiguous animal scenarios (Lester et al. 2011). However, child and adolescent CBM-I studies do not uniformly find effects on mood (Cristea et al. 2015; Lester et al. 2011). Some studies have reported effects on mood in particular participant sub-groups only (Lau et al. 2011), or only following exposure to a laboratory stressor (Lau et al. 2012). In the current study, impact on positive affect was small, which may be due to the use of a healthy rather than clinical sample whose levels of positive affect may already be high. Further studies are needed to understand these effects and to explore the contribution of mental imagery specifically (Lau and Pile 2015).

The current study found no differential effects of imagery training valence on negative mood as measured using fear and sadness items from the PANAS. This result potentially reflects floor effects in our non-clinical sample, consistent with previous studies (Rohrbacher et al. 2014). To evaluate this suggestion, future studies should include baseline measures of current depression and anxiety symptoms, and a greater variety of participants.

We found no moderating effects of perspective on positive mood in the current study, despite predictions of greater mood change following field than observer perspective imagery. As this adolescent imagery training study is the first of its kind, this finding warrants further investigation, particularly given that experimental investigations of the impact of imagery perspective in the adult literature have also produced mixed findings (e.g. Holmes et al. 2008a, b; Nelis et al. 2013).

### Increase in Pleasantness Ratings of Picture Stimuli Following Positive Imagery Training for Field But Not Observer Perspective Imagery

Pleasantness ratings of picture stimuli provide a measure of interpretation bias. As predicted, results indicate greater increases in pleasantness ratings following positive than mixed imagery for field perspective imagery. This pattern did not differ significantly according to whether or not images had previously been seen in the context of the imagery generation task, thus indicating generalization of the effect beyond the trained stimuli. Importantly, greater pleasantness ratings following positive than mixed field perspective imagery were not simply a function of state mood, which was equivalent across groups at the assessment. Previous studies that have used picture-word imagery training procedures in adults report similar effects of valence on ratings of picture stimuli (Holmes et al. 2008b; Pictet et al. 2011). Results from the current study extend these findings, suggesting positive picture-word imagery training is effective in modifying how healthy adolescents interpret ambiguous visual scenes after their mood has returned to baseline. Critically, this effect was only seen when imagery was generated from a first (not third) person perspective. This result is broadly consistent with previous work suggesting the perspective taken in mental imagery modulates its impact on subsequent processing (Berntsen and Rubin 2006; Libby and Eibach 2002). The current result is also consistent with correlational evidence that spontaneous adoption of an observer perspective is associated with reduced emotional impact of recalling an affectively-laden event in male participants aged 11–20 years (White et al. 2015).

Our finding of an effect of imagery perspective on interpretation bias in the absence of predicted effects on immediate mood requires following up in further studies. Notwithstanding these outstanding questions, the current finding suggests that explicitly instructing adolescent participants on how to adopt a field perspective may enhance the effects of positive imagery training on later positive cognitive processing.

### Fewer Negative Interpretations on a Novel Scrambled Sentence Task Following Positive Imagery Training for Field But Not Observer Perspective Imagery

On a novel SST measure of interpretation bias, participants showed lower negativity scores following positive than mixed imagery generated from a field but not an observer perspective. This pattern of results is consistent with results from the pleasantness ratings task measure of interpretation bias. It is possible that field perspective imagery encourages more self-relevant processing of the positive training material, and thus has a greater subsequent impact than observer perspective imagery on processing of other self-relevant information (such as the sentences in the SST). Whereas stimuli in the pleasantness rating task were similar in format to stimuli used in the imagery training task, SST stimuli were quite different in format. As such, this converging result from the SST supports the notion that the imagery training procedure effects transfer to tasks with a different format than the initial training.

While several studies in adults have shown the impact of imagery training on the SST (Blackwell and Holmes 2010; Holmes et al. 2009; Lang et al. 2012), to our knowledge, this is the first study to report effects of positive imagery training on SST negativity in adolescents. Provisionally, explicit instruction to adolescent participants on how to adopt a field versus observer perspective may enhance the effects of imagery training valence on interpretation bias. However, it should be noted that effect sizes were small.

The SST used in the current study consisted of adolescent-appropriate statements relating to concerns in anxiety and social anxiety. Studies have shown that negative interpretation bias is observed in anxious and depressed populations (Hertel and Mathews 2011; Orchard et al. 2015), and that scores on an SST predict future depression (Rude et al. 2002, 2003, 2009; Blackwell et al. 2015). Therefore, an impact of imagery training on this measure of cognitive bias is promising. In adolescence, concerns regarding peer evaluation are prominent. In the current study, a large proportion of the imagery generation stimuli suggested social situations involving peers, and we developed the novel adolescent SST with a view to picking up on differences in cognitive processing that related specifically to adolescent social concerns. The result we obtained using this measure highlights our novel SST as a potentially useful tool to investigate negative interpretation bias in adolescents specifically.

### No Effects on Computerised Recognition Task Measure of Interpretation Bias

Contrary to predictions, we found no effects of imagery valence or perspective on an adapted version of a computerised recognition task measure of interpretation bias. This widely used measure has previously been administered in several adolescent CBM-I studies using a verbal scenario-based training paradigm (e.g. Lothmann et al. 2011; Lau et al. 2012; Fu et al. 2013), and has shown sensitivity to training effects. However, the training in these studies closely matches the format and content of the CRT, and thus such verbal scenario-based paradigms may be better-placed to induce changes on this measure.

### Applicability

In the current study, the majority of training scenarios depicted common everyday situations. Rather than being homogenously positive, picture stimuli depicted an ambiguous scene. The participant must then create something positive from this ambiguity, by combining it with the positive word caption via mental imagery. When applied in a clinical context over multiple sessions of training, in theory the training should lead to an increased tendency to imagine positive resolutions in the context of similar ambiguous situations in everyday life.

In a previous imagery training study in adults, participants reported experiencing involuntary memories of positive training stimuli, triggered by real-life situations that resemble the training stimuli (Blackwell and Holmes 2010). This suggests positive imagery may become infused into everyday life, although this phenomenon has not been systematically investigated. Interestingly, some have argued the possibility that a mixture of positive and negative resolutions could have benefits and increase cognitive flexibility (cf. Blackwell et al. 2015), and simply imagining positive possibilities is unlikely to provide a panacea.

Within a therapeutic context, positive imagery training is perhaps best thought of as a potential adjunct to other approaches (e.g. CBT, cf. Williams et al. 2013, 2015), rather than a standalone treatment. While imagery seems useful to enable individuals to envision positive resolutions of ambiguity, doing so when thinking about negative events may be less useful—due to the mood-amplifying effect of imagery in that case (i.e. negative imagery). As such, the current study focused on the promotion of positive interpretations via positive mental imagery. Real life, however, contains a backdrop of ambiguity that is less homogenous. Suggestions have been proposed for how mental imagery may be incorporated into everyday life (Holmes et al. 2016a, b; Pearson et al. 2015), and imagery-based psychotherapy techniques have been discussed in detail (Holmes et al. 2007, 2016a, b). Furthermore, there is evidence that imagery training may be useful in a wider variety of non-therapeutic contexts too, such as reducing prejudice (West et al. 2011).

## Summary

Results show that generating positive mental imagery resulted in increases in positive mood and interpretations. Effects on interpretation bias were not attributable to affective carryover from the mental imagery training manipulation (i.e., mood priming), since a filler task equalised mood prior to administration of measures of interpretation bias. The potentially beneficial effects of positively valenced mental imagery on two measures of interpretation bias occurred when imagery was generated from a field (first person) perspective, and did not occur when mental imagery was generated from an observer (third person) perspective. These findings highlight the impact of positive mental imagery generation on mood and cognition in male adolescents. They also suggest that explicitly instructing male adolescent participants in how to adopt a field perspective in mental imagery may enhance the impact of positive imagery training on interpretation bias. Future research should extend these findings to female adolescents and clinical groups, as the current findings are not generalizable to these populations.

Lifetime mental health problems commonly have their inception during adolescence. As such, early intervention is crucial. Understanding the cognitive factors that cause, maintain, or indeed protect against psychopathology is a promising avenue for research. Findings from the current study have implications for understanding cognitive mechanisms and moderators underlying the relationship between mental imagery, mood and cognitive processing in adolescents (Burnett Heyes et al. 2013). Potentially, mental imagery-based techniques may be valuable as a clinical treatment strategy in child and adolescent groups, and as a preventative cognitive “vaccine” to boost resilience in those at risk of emotional disorders (Holmes et al. 2009).

## Electronic supplementary material

Below is the link to the electronic supplementary material.

Supplementary material 1 (DOCX 81 kb)

## Footnotes

1 When SAS-A total score was not included as a covariate, main effect of Valence was [*F*(1,56) = 2.31, *p*_1-tailed_ = .067, *ηp* ^2^ = .040] and interaction between Valence and Perspective [*F*(1,56) = 2.67, *p*_1-tailed_ = .054, *ηp* ^2^ = .045].
