# Supplementary material for: Mental Imagery-Based Training to Modify Mood and Cognitive Bias in Adolescents: Effects of Valence and Perspective
Source: Cognit Ther Res. 2016 Aug 8;41(1):73–88. doi: 10.1007/s10608-016-9795-8 (PMC5306169; doi:10.1007/s10608-016-9795-8)
Supplement: Supplementary file 1 — Supplementary material 1 (DOCX 81 kb) [file 10608_2016_9795_MOESM1_ESM.docx]

**Table S1. Adolescent anxiety and social anxiety scrambled sentence stimuli used in the novel Scrambled Sentences Task (SST).** Forty stimuli were created resulting in two different versions of the SST. See Method section for details.

| Scrambled sentence | Positive or negative resolution |
| --- | --- |
| practice often guitar I my conversations | I often practice my guitar/conversations |
| people dislike new enjoy meeting I | I enjoy/dislike meeting new people |
| aches tummy I never have often | I have tummy aches never/often |
| am energetic I person indecisive an | I am an energetic/indecisive person |
| others to appear sensible I foolish | I appear sensible/foolish to others |
| right say things wrong I the | I say the right/wrong things |
| poorly well people of me speak | People speak well/poorly of me |
| person am a nervous I cheerful | I am a cheerful/nervous person |
| I so friends mistakes make many | I make so many friends/mistakes |
| with people other easy uneasy I'm | I'm easy/uneasy with other people |
| I always off sick am never | I am never/always off sick |
| my people laugh jokes at clothes | People laugh at my jokes/clothes |
| scary new fine people approaching is | Approaching new people is fine/scary |
| daydream I worry about future the | I daydream/worry about the future |
| relax fully love struggle to I | I love/struggle to fully relax |
| I social most avoid occasions enjoy | I enjoy/avoid most social occasions |
| sleep don't I well very do | I do/don't sleep very well |
| other may please I must people | I may/must please other people |
| mistakes my notice people will talents | People will notice my talents/mistakes |
| engagements I social usually endure enjoy | I usually enjoy/endure social engagements |
| new people with confident I'm nervous | I'm confident/nervous with new people |
| I upset at home feel relaxed | I feel relaxed/upset at home |
| read hours for sometimes I worry | I sometimes read/worry for hours |
| I very happy often feel tense | I often feel very happy/tense |
| a winner worrier I think I'm | I think I’m a winner/worrier |
| relaxed with tense I'm children older | I'm relaxed/tense with older children |
| boring people nice I think am | People think I am nice/boring |
| good make I impression bad | I make a good/bad impression |
| avoid usually social events I attend | I usually attend/avoid social events |
| am I with careful carefree others | I am carefree/careful with others |
| feels body achy my usually good | My body usually feels good/achy |
| avoid face I worst my fears | I face/avoid my worst fears |
| to talking like I people dislike | I like/dislike talking to people |
| I all win cry time the | I win/cry all the time |
| me approve people other of disapprove | Other people approve/disapprove of me |
| upsetting occasions I social find enjoyable | I find social occasions enjoyable/upsetting |
| involved films jumpy in I get | I get involved/jumpy in films |
| sounds so laugh happy ridiculous my | My laugh sounds so happy/ridiculous |
| always edge am I form on | I am always on form/edge |
| full so feels sore my tummy | My tummy feels so full/sore |

**Figure S1. Flow of participants through experimental sessions.** PANAS = Positive and negative affect schedule.

**
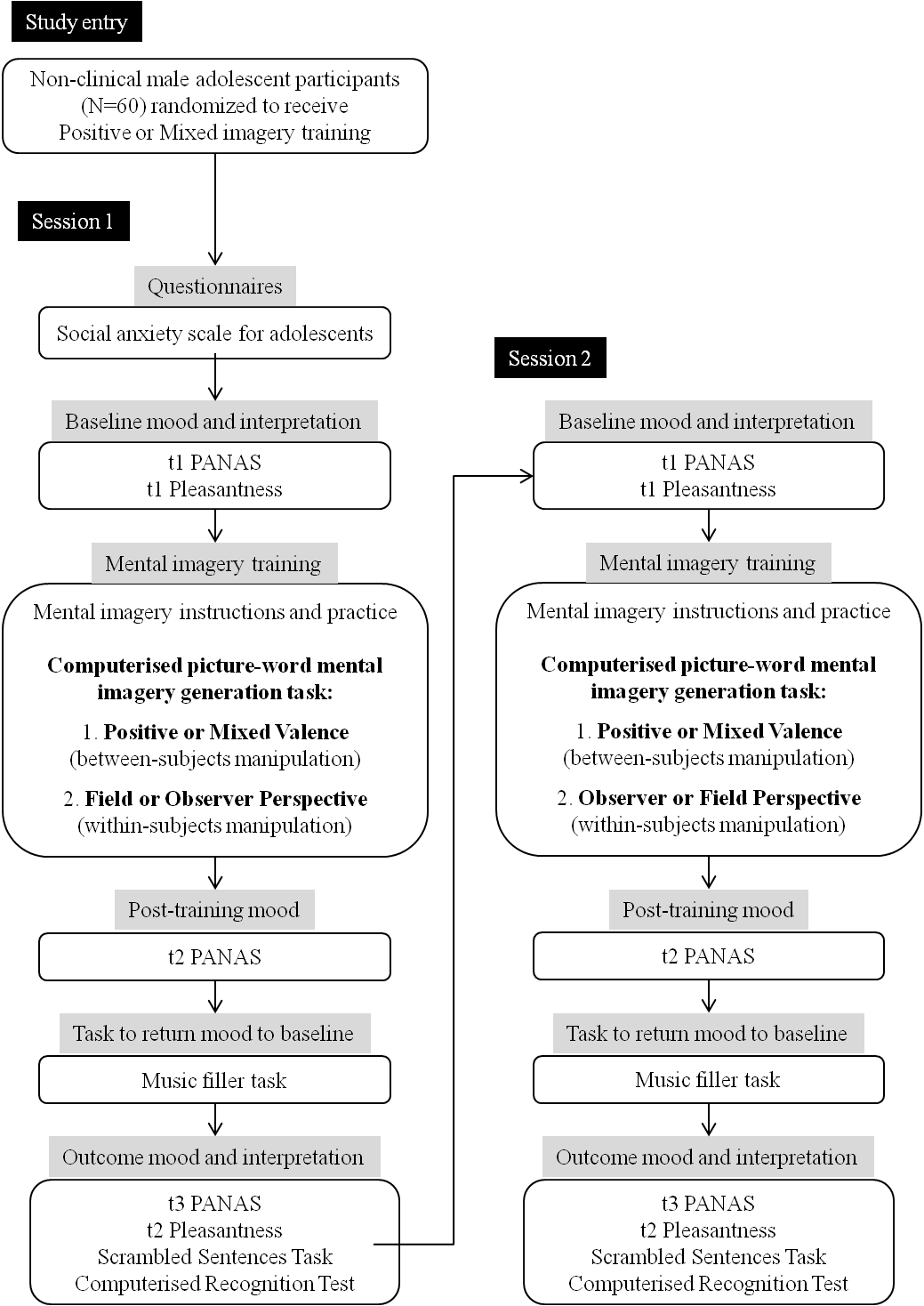
**
